# Clinical Characteristics, Treatment Patterns, and Survival Outcomes of Right-Sided RAS/RAF Wild-Type Metastatic Colorectal Cancer: A Real-World Multicenter Cohort Study

**DOI:** 10.3390/clinpract16090158

**Published:** 2026-08-24

**Authors:** Nur Deniz Yildiz, Çağatay Arslan, İlker Nihat Okten, Umut Kefeli, Mahmut Emre Yildirim, Nuri Karadurmus, Tuba Baydas, Bülent Karabulut, Irfan Cicin, Cemil Bilir, Melike Ozcelik, Timucin Cil, Sinemis Celik, Oktay Bozkurt, Hakan Harputluoglu, Bala Başak Oven, Mehmet Artaç, Hacı Mehmet Türk, Ahmet Alacacıoğlu, Mahmut Gumus, Şuayib Yalcin

**Affiliations:** 1 Department of Molecular Oncology, Istanbul Medeniyet University, 34700 Istanbul, Türkiye; 2 Department of Medical Oncology, Faculty of Medicine, İzmir Ekonomi University, Medical Park Hospital, 35330 İzmir, Türkiye; 3 Department of Medical Oncology, Istanbul Medeniyet University, Göztepe Şehir Hospital, 34722 Istanbul, Türkiye; ilker.okten@medeniyet.edu.tr (İ.N.O.); tuba.baydas@medeniyet.edu.tr (T.B.); mahmut.gumus@medeniyet.edu.tr (M.G.); 4 Department of Oncology, Faculty of Medicine, Kocaeli University, 41001 Kocaeli, Türkiye; 5 Medical Park Pendik Hospital, 34899 Istanbul, Türkiye; 6 Department of Medical Oncology, University of Health Sciences, Gülhane Training and Research Hospital, 06010 Ankara, Türkiye; 7 Acıbadem Kent Hospital, 35620 İzmir, Türkiye; 8 Department of Medical Oncology, Istinye University Liv Hospital Topkapı, 34475 Istanbul, Türkiye; 9 Department of Medical Oncology, Istinye University, 34475 Istanbul, Türkiye; 10 Department of Medical Oncology, Ümraniye Education and Research Hospital, 34764 Istanbul, Türkiye; 11 Adana City Hospital, Saglık Bilimleri University, 01230 Adana, Türkiye; 12 Medical Park Istanbul Oncology Hospital, 34746 Istanbul, Türkiye; 13 Department of Medical Oncology, Erciyes University Hospital, 38039 Istanbul, Türkiye; oktaybozkurt@erciyes.edu.tr; 14 Department of Medical Oncology, Memorial Bahcelievler Hospital, 34180 Istanbul, Türkiye; 15 Department of Medical Oncology, Istanbul Yeditepe University Hospital, 34755 Istanbul, Türkiye; 16 Department of Medical Oncology, Necmettin Erbakan University Meram Medicine Faculty, 42090 Konya, Türkiye; 17 Department of Medical Oncology, Bezmialem Vakıf University Hospital, 34093 Istanbul, Türkiye; 18 Department of Medical Oncology, Izmir Katip Celebi University, 35620 Izmir, Türkiye; 19 Department of Medical Oncology, Hacettepe University, 06230 Ankara, Türkiye

**Keywords:** metastatic colorectal cancer, right-sided colon cancer, RAS wild-type, anti-EGFR therapy

## Abstract

**Background**: Right-sided metastatic colon cancer represents a clinically distinct subgroup with inferior outcomes and uncertain optimal biologic treatment selection, even among patients with RAS wild-type disease. Real-world data focusing specifically on right-sided RAS wild-type metastatic colon cancer remain limited. This study aimed to describe clinicopathologic characteristics, metastatic patterns, treatment approaches, and survival outcomes in this population using a national multicenter registry. **Methods**: This retrospective multicenter cohort study was conducted using data from the ONKO-KOLON Türkiye registry. Patients with pathologically confirmed KRAS/NRAS wild-type metastatic colorectal cancer and available primary tumor localization were evaluated. The main analytic cohort included patients with right-sided metastatic colon cancer, defined as right colon or transverse colon tumors. Left-sided colon cancer patients were used as a contextual comparator, while rectal cancer patients were excluded from sidedness-based colon comparisons. Survival outcomes were estimated using the Kaplan–Meier method, and prognostic factors were evaluated using Cox regression analyses. **Results**: Among 1079 patients in the source cohort, primary tumor localization was available in 1065 patients. Of these, 213 had right-sided colon cancer, 464 had left-sided colon cancer, and 388 had rectal cancer. In the right-sided cohort, median age was 61.5 years, 64.3% were male, and 66.7% had synchronous/de novo metastatic disease. Liver metastasis was the most common metastatic site (63.4%), followed by lymph node (31.0%), lung (21.1%), and peritoneal metastases (15.5%). First-line anti-VEGF-based treatment was used in 46.0% of patients, while anti-EGFR-based treatment was used in 40.8%. Among evaluable patients, the objective response rate was 46.8% and the disease control rate was 79.2%. Median progression-free survival was 10.0 months, and median overall survival was 24.0 months. In unadjusted exploratory analysis, median OS was 27.0 months with anti-EGFR-based treatment and 17.0 months with anti-VEGF-based treatment (log-rank *p* = 0.022), whereas median PFS was 10.0 months in both groups. In an extended covariate-adjusted multiple-imputation sensitivity model, the anti-EGFR OS estimate did not meet statistical significance (adjusted HR 0.66, 95% CI 0.43–1.00; *p* = 0.051). In a secondary contextual comparison, median overall survival was shorter in the right-sided than in the left-sided colon cancer group (24.0 vs. 28.0 months; HR 1.47, 95% CI 1.19–1.82; *p* < 0.001), whereas progression-free survival did not differ significantly. **Conclusions**: This study provides a descriptive account of metastatic patterns, treatment approaches, and outcomes in a dedicated right-sided RAS wild-type metastatic colon cancer cohort. The unadjusted OS difference between biological treatment groups was not confirmed after measured covariate adjustment and should not be interpreted as evidence of comparative treatment effectiveness. Because molecular profiling was incomplete, this study cannot identify biomarker-defined treatment subgroups. Prospective studies with complete, predefined molecular characterization are needed before molecularly informed treatment selection hypotheses can be evaluated in this population.

## 1. Introduction

Colorectal cancer remains one of the most common malignancies worldwide and is a major cause of cancer-related mortality [1,2]. In 2022, more than 1.9 million new colorectal cancer cases and more than 900,000 deaths were estimated globally, highlighting the continuing clinical burden of this disease. A substantial proportion of patients either present with metastatic disease or develop metastases during follow-up, making treatment optimization in metastatic colorectal cancer (mCRC) a central issue in gastrointestinal oncology.

Over the past two decades, the treatment landscape of mCRC has evolved considerably with the integration of oxaliplatin- and irinotecan-based chemotherapy backbones, anti-VEGF therapy, anti-EGFR monoclonal antibodies, and molecularly guided treatment strategies [2,3,4,5,6,7,8]. In patients with RAS wild-type disease, anti-EGFR agents such as cetuximab and panitumumab have become important therapeutic options [3,4,5]. However, the benefit of anti-EGFR therapy is strongly influenced by primary tumor location, and tumor sidedness is now recognized as both a prognostic and predictive factor in RAS wild-type mCRC [9,10,11].

Right-sided colon cancers differ from left-sided tumors in embryologic origin, molecular characteristics, metastatic behavior, and clinical outcomes [12,13,14]. Pooled analyses of randomized studies have shown that patients with right-sided RAS wild-type mCRC generally have inferior survival outcomes compared with those with left-sided tumors [9,10,11,15]. These analyses also demonstrated that the predictive value of primary tumor sidedness is particularly relevant for anti-EGFR therapy, with the clearest benefit observed in left-sided tumors.

Current treatment recommendations therefore incorporate sidedness into first-line decision-making [16,17]. For RAS wild-type and BRAF wild-type left-sided mCRC, chemotherapy plus anti-EGFR therapy is generally preferred when clinically appropriate. In contrast, for right-sided RAS wild-type tumors, chemotherapy with or without bevacizumab is commonly preferred, although anti-EGFR-based therapy may still be considered in selected clinical scenarios where tumor shrinkage or conversion to resectability is an important goal [16,17,18].

Although the prognostic and predictive importance of primary tumor sidedness is well established [9,10,11,15], existing randomized-trial subgroup analyses and pooled studies do not fully describe how patients with right-sided RAS wild-type metastatic colon cancer present and are managed in routine clinical practice. These studies generally include highly selected trial populations and frequently evaluate sidedness in cohorts containing both colon and rectal primary tumors [9,10,11,15]. Consequently, information remains limited regarding metastatic distribution, real-world biologic treatment selection, treatment response, and survival outcomes in a dedicated right-sided colon cancer cohort [19]. National multicenter data are particularly limited in Türkiye, where treatment selection in routine practice may also be influenced by reimbursement conditions, drug availability, physician preferences, and institutional practice patterns [2,16,17,19].

The present study was therefore designed primarily as a descriptive real-world registry analysis rather than as a comparative-effectiveness study. Using the ONKO-KOLON Türkiye registry, we aimed to characterize the clinicopathologic features, metastatic patterns, first-line treatment strategies, treatment response, and survival outcomes of patients with right-sided RAS wild-type metastatic colon cancer treated in routine practice. Unlike many previous sidedness analyses, rectal cancers were excluded from the colon-sidedness comparison. Patients with left-sided colon cancer were included only as a contextual comparator, and comparisons between biological treatment strategies were considered exploratory because treatment allocation was not randomized. The principal contribution of this analysis is therefore a detailed national description of clinicopathologic presentation, metastatic distribution, and routine-care management in a dedicated right-sided colon cancer cohort, rather than a new evaluation of the established prognostic effect of sidedness or of the comparative effectiveness of biological agents.

## 2. Methods and Materials

### 2.1. Study Design and Data Source

This retrospective multicenter cohort study was conducted as a secondary analysis of the ONKO-KOLON Türkiye registry, a nationwide real-world registry of patients with RAS wild-type metastatic colorectal cancer treated at 28 participating oncology centers across Türkiye [19]. Medical records were collected for patients diagnosed with metastatic colorectal cancer between January 2016 and April 2019. The present analysis used the registry dataset containing demographic, clinicopathologic, molecular, treatment-related, response, and survival information derived from routine clinical practice.

At each participating center, relevant data were retrospectively abstracted from patients’ medical records. Recorded variables included demographic characteristics Eastern Cooperative Oncology Group (ECOG) performance status, primary tumor location, histopathologic and molecular findings, metastatic presentation and distribution, systemic treatment, treatment response, disease progression, and survival status. Patients were followed through routine clinical practice until death or their last documented clinical follow-up.

Before analysis, the consolidated database was checked for duplicate records, eligibility, internal consistency, invalid category codes, and implausible dates. Identified discrepancies were queried with the participating centers and corrected where possible. No centralized pathological or radiological review and no external source-data verification were performed; pathological findings, molecular testing, radiological response assessment, and follow-up procedures were based on routine practice at the participating institutions.

Because the registry was retrospective, the availability of molecular, histopathologic, tumor-marker, and performance-status variables differed among patients. The registry did not contain a structured field documenting the patient-level reason for each absent value. Consequently, it was not possible to distinguish reliably whether individual missing observations reflected testing that was not performed, results that were not documented, or information that could not be retrieved from the source medical record. In particular, BRAF and MSI testing was not uniform across participating centers during the study period, while ECOG performance status, histopathologic variables, and tumor-marker results were not consistently documented. Variable-specific denominators and missing-data frequencies are reported in the Results and Supplementary Table S1. Missingness was also examined according to first-line biological treatment group. No missing values were assumed to be negative or normal.

For the present study, the main analytic cohort consisted of patients with right-sided RAS wild-type metastatic colon cancer. Patients with left-sided colon cancer were included only as a contextual comparator, while patients with rectal cancer were excluded from the colon-sidedness comparison. The registry was not population-based and included patients treated at participating oncology centers. Therefore, although its multicenter structure reflects routine practice across different institutions in Türkiye, it should not be interpreted as fully representative of every patient with metastatic colorectal cancer in the country.

### 2.2. Study Population

The source cohort consisted of patients with pathologically confirmed metastatic colorectal cancer and documented KRAS/NRAS wild-type status. Patients were eligible for the present analysis if they had metastatic colon cancer and available information on primary tumor location.

Right-sided colon cancer was defined according to the registry-derived sidedness variable available in the de-identified analytic dataset. The source registry grouped tumors recorded as right colon or transverse colon within the right-sided category, and this prespecified classification was retained for the primary analysis. However, the analytic dataset did not retain a separate patient-level variable that reliably identified transverse colon tumors. Therefore, a sensitivity analysis excluding transverse colon cases could not be performed without potentially introducing misclassification. Accordingly, primary tumor subsite counts are not reported, and all analyses were performed using the registry-derived right-sided category.

Patients with missing primary tumor localization were excluded from sidedness-based analyses. For secondary comparative analyses, left-sided colon cancer was defined according to the registry-coded left colon category. Rectal tumors were excluded from this comparator analysis to avoid mixing colon and rectal cancer biology, treatment strategies, and prognosis.

BRAF mutation status was recorded when available and analyzed descriptively. Because BRAF testing was not uniformly available across the cohort, BRAF mutation status was not used as an inclusion or exclusion criterion.

**Figure 1:**
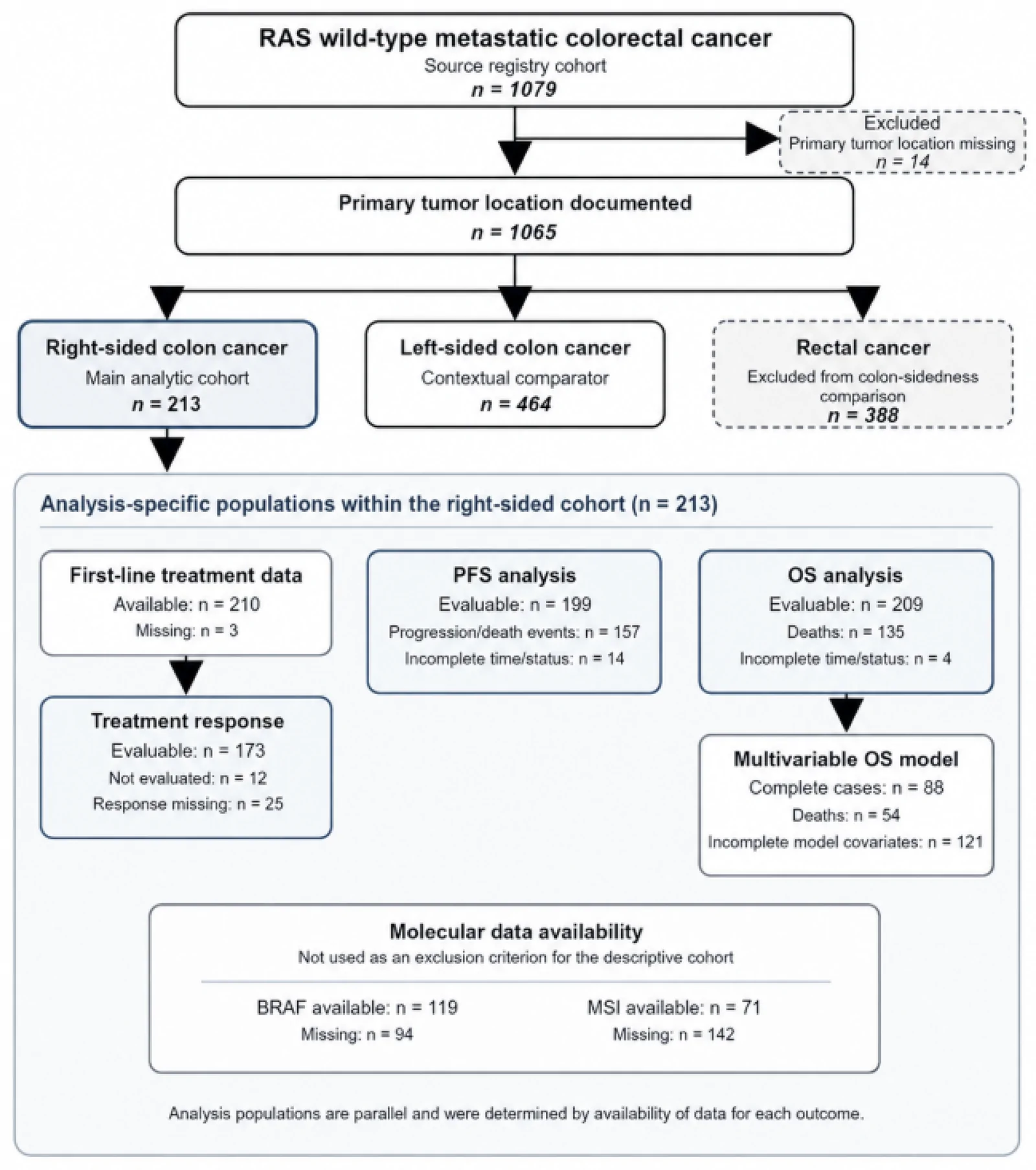
Patient selection and analysis-specific populations. PFS, progression-free survival; OS, overall survival; MSI, microsatellite instability.

Patient selection proceeded hierarchically. The source registry included 1079 patients with documented RAS wild-type metastatic colorectal cancer. Fourteen patients without documented primary tumor localization were excluded from sidedness-based classification, leaving 1065 patients with known tumor location. Of these, 388 patients with rectal cancer were excluded from the colon-sidedness comparison, 464 patients with left-sided colon cancer formed the contextual comparator group, and 213 patients with right-sided colon cancer constituted the main analytic cohort. All 213 patients were included in the descriptive clinicopathologic analyses. Analysis-specific populations were subsequently defined according to data availability: first-line treatment data were available for 210 patients, response was evaluable in 173 patients, PFS was evaluable in 199 patients, and OS was evaluable in 209 patients. Missing BRAF or MSI results did not lead to exclusion from the main cohort; these variables were examined using available-case denominators. The multivariable OS model was performed as a complete-case analysis. The complete cohort-selection process and analysis-specific denominators are presented in Figure 1.

### 2.3. Variables and Definitions

Extracted variables included age at diagnosis, sex, body mass index, smoking status, Eastern Cooperative Oncology Group performance status, primary tumor localization, de novo versus metachronous metastatic presentation, histological grade, mucinous histology, microsatellite instability status, BRAF mutation status, baseline carcinoembryonic antigen level, baseline carbohydrate antigen 19-9 level, sites and number of metastatic organs, primary tumor resection status, metastasectomy status, first-line systemic treatment regimen, biological agent use, chemotherapy backbone, treatment response, maintenance treatment, second-line treatment, progression-free survival, and overall survival. Baseline CEA was categorized as ≤5 versus >5 ng/mL, and baseline CA19-9 was categorized as ≤35 versus >35 U/mL, according to the cutoffs used in the registry dataset.

Metastatic presentation was categorized as de novo metastatic disease or metastatic recurrence after initial treatment for earlier-stage disease. Metastatic sites were categorized as liver, lung, peritoneum, lymph node, and other sites according to registry records. Liver-only metastasis and the number of metastatic organs were additionally assessed where available.

Metastasectomy was defined as surgical removal of metastatic lesion(s) at any time during the disease course, regardless of whether the procedure was performed with curative or cytoreductive intent. This variable was included as a clinical prognostic factor but was not the central focus of the present analysis.

### 2.4. Treatment Variables

First-line systemic treatment was categorized according to the chemotherapy backbone and the biological agent used. Chemotherapy backbone was classified as oxaliplatin-based, irinotecan-based, triplet chemotherapy, fluoropyrimidine-only/other, or missing/unclear, based on the regimen recorded in the registry.

Biological treatment was categorized as anti-EGFR-based treatment, anti-VEGF-based treatment, chemotherapy without biological agent, or missing/unclear. Anti-EGFR treatment included cetuximab- or panitumumab-containing regimens. Anti-VEGF treatment included bevacizumab-containing regimens.

For descriptive purposes, first-line biological agents were also evaluated separately as panitumumab, cetuximab, and bevacizumab. The registry recorded the date of first-line treatment initiation, whether maintenance treatment was administered, the maintenance-treatment start date and regimen, and whether any second-line systemic therapy was administered. However, it did not systematically capture the number of treatment cycles, cumulative dose or dose intensity, dose reductions or treatment delays, the actual date or reason for first-line discontinuation, total treatment duration, or the specific regimens and sequencing of subsequent treatment lines. Accordingly, these exposure measures could not be analyzed, and PFS was not used as a surrogate for treatment duration. Because maintenance treatment and receipt of second-line therapy may be influenced by treatment response, survival duration, and patient selection, these variables were summarized descriptively and were not interpreted as causal treatment effects.

### 2.5. Response and Survival Outcomes

Treatment response to first-line therapy was recorded according to routine clinical and radiological assessments documented in the registry and categorized as complete response, partial response, stable disease, progressive disease, not evaluable, or missing. Objective response rate was defined as the proportion of evaluable patients achieving complete or partial response. Disease control rate was defined as the proportion of evaluable patients achieving complete response, partial response, or stable disease.

The primary survival endpoint was overall survival among patients with right-sided RAS wild-type metastatic colon cancer. Overall survival was defined as the time from initiation of first-line systemic therapy for metastatic disease to death from any cause or last known follow-up.

Progression-free survival was defined as the time from initiation of first-line systemic therapy for metastatic disease to documented disease progression, death from any cause, or last follow-up, whichever occurred first. Disease progression was determined according to routine clinical and radiological follow-up as recorded in the registry.

Secondary endpoints included progression-free survival, objective response rate, disease control rate, first-line treatment patterns, metastatic site distribution, and prognostic factors associated with survival outcomes within the right-sided cohort. A secondary contextual analysis compared selected clinicopathologic characteristics and survival outcomes between right-sided and left-sided colon cancer patients.

### 2.6. Statistical Analysis

Multivariable Cox regression modeling was undertaken when the number of events and the availability of covariate data were considered sufficient to support estimation. Variables with clinical relevance and/or *p* values below 0.10 in univariable analysis were considered for inclusion. The primary multivariable OS model included ECOG performance status, primary tumor resection, metastasectomy, first-line biological treatment category, and chemotherapy backbone. This primary model was performed using complete cases. The extent of missingness was summarized for each key variable, and patients included in the complete-case model were compared with those excluded using the Mann–Whitney U test, chi-square test, or Fisher’s exact test, as appropriate. Overall survival between complete-case and excluded patients was additionally compared using Kaplan–Meier estimates and the log-rank test.

Median follow-up from initiation of first-line systemic therapy was estimated using the reverse Kaplan–Meier method, in which deaths were treated as censored observations and patients alive at last contact as events. Follow-up was summarized for the combined right- and left-sided colon cohort, separately by tumor sidedness, and within the first-line anti-EGFR and anti-VEGF treatment groups.

As a sensitivity analysis, multiple imputation by chained equations with predictive mean matching was performed among patients with evaluable OS, classifiable anti-EGFR or anti-VEGF treatment exposure, and a classifiable oxaliplatin- or irinotecan-based chemotherapy backbone. Treatment categories outside these modeled contrasts were considered structurally ineligible and were not imputed. Fifty imputed datasets were generated for missing ECOG performance status, primary tumor resection status, and age as an auxiliary predictor. The imputation model incorporated age, sex, metastatic presentation, metastatic burden and sites, metastasectomy, biological treatment group, chemotherapy backbone, OS time, and death status. Multiple imputation was performed for the primary OS model; the exploratory PFS model was analyzed using complete cases. Cox regression estimates were pooled across imputed datasets according to Rubin’s rules. Primary analyses were performed using SPSS version 26.0; missing-data assessments and the multiple-imputation sensitivity analysis were performed using Python version 3.12 with statsmodels version 0.14.6.

To reduce potential immortal-time bias in the surgery-related survival analyses, additional univariable time-dependent Cox sensitivity models were fitted for primary tumor resection and metastasectomy. Procedures performed before first-line treatment initiation were classified as present from time zero, whereas procedures performed after treatment initiation were modeled as time-dependent exposures, with patients considered unexposed until the recorded procedure date. Patients with a documented procedure but unavailable procedure timing were excluded from the corresponding time-dependent analysis.

### 2.7. Ethical Considerations

The study was conducted in accordance with the principles of the Declaration of Helsinki. The ONKO-KOLON Türkiye registry was approved by the Istanbul Medeniyet University Göztepe Training and Research Hospital Clinical Research Ethics Committee. Because of the retrospective design and use of anonymized clinical data, the requirement for informed consent was waived.

## 3. Results

### 3.1. Patient Selection and Cohort Definition

The source cohort included 1079 patients with RAS wild-type metastatic colorectal cancer. Primary tumor localization was available for 1065 patients, while 14 patients with missing localization data were excluded from sidedness-based classification. Among patients with known tumor localization, 213 had right-sided colon cancer, 464 had left-sided colon cancer, and 388 had rectal cancer. The 213 patients with right-sided colon cancer constituted the main analytic cohort; the left-sided colon cancer group was retained as a contextual comparator, whereas patients with rectal cancer were excluded from the colon-sidedness comparison.

Within the right-sided cohort, first-line treatment data were available for 210 patients and missing for three patients. Among the 210 patients with documented first-line treatment, 173 were evaluable for treatment response; 12 were explicitly recorded as not evaluated, and response information was missing for 25. PFS was evaluable in 199 patients, including 157 progression or death events, while 14 patients were excluded from the PFS analysis because of incomplete survival-time or event-status information. OS was evaluable in 209 patients, including 135 deaths, while four patients were excluded because of incomplete survival-time or vital-status information. BRAF and MSI results were available for 119 and 71 patients, respectively, and missing molecular results did not result in exclusion from the primary descriptive cohort. The complete-case multivariable OS model included 88 patients and 54 deaths. The cohort-selection process and analysis-specific populations are summarized in Figure 1.

### 3.2. Baseline Clinicopathologic Characteristics of the Right-Sided Cohort

The baseline characteristics of the right-sided cohort are summarized in Table 1. The median age was 61.5 years (range, 26–84), and 137 patients (64.3%) were male. Most patients had synchronous/de novo metastatic disease (66.7%). ECOG performance status was available in 128 patients; among them, 110 patients had ECOG 0–1 and 18 patients had ECOG 2–4.

BRAF status was available in 119 patients; 9 patients had BRAF-mutant disease, while 110 patients were BRAF wild-type. MSI status was available in 71 patients; 15 patients were MSI-H and 56 patients were MSS. Primary tumor resection had been performed in 161 patients (75.6%), and 30 patients (14.1%) underwent metastasectomy.

### 3.3. Metastatic Distribution

Within the right-sided cohort, the most frequent metastatic site was the liver, observed in 135 patients (63.4%), followed by lymph node metastasis in 66 patients (31.0%), lung metastasis in 45 patients (21.1%), and peritoneal metastasis in 33 patients (15.5%). Liver-only metastasis was present in 83 patients (39.0%). Most patients had involvement of a single metastatic organ (65.3%) (Table 2).

### 3.4. First-Line Treatment Patterns and Treatment Response

First-line systemic treatment data were available in 210 of 213 right-sided patients. The most common first-line treatment category was doublet chemotherapy plus anti-VEGF therapy, used in 98 patients (46.0%), followed by doublet chemotherapy plus anti-EGFR therapy in 87 patients (40.8%). Chemotherapy doublet without a biological agent was used in 21 patients (9.9%), while 4 patients (1.9%) received triplet-based treatment.

Regarding individual biological agents, 102 patients (47.9%) received bevacizumab-containing therapy, comprising 98 doublet-plus-anti-VEGF regimens and 4 triplet-plus-bevacizumab regimens. Cetuximab-containing therapy was administered to 51 patients (23.9%), and panitumumab-containing therapy to 36 patients (16.9%). Oxaliplatin-based chemotherapy was used in 129 patients (60.6%), while irinotecan-based chemotherapy was used in 67 patients (31.5%).

Maintenance-treatment status was documented for 207 patients, of whom 54/207 (26.1%) received maintenance therapy. Among these 54 patients, the recorded maintenance regimens were capecitabine plus bevacizumab in 16 patients (29.6%), capecitabine alone in 10 (18.5%), 5-fluorouracil/leucovorin plus bevacizumab in 8 (14.8%), 5-fluorouracil/leucovorin plus panitumumab in 8 (14.8%), 5-fluorouracil/leucovorin plus cetuximab in 8 (14.8%), cetuximab alone in 3 (5.6%), and capecitabine plus cetuximab in 1 (1.9%). Second-line treatment status was documented for 208 patients, of whom 117/208 (56.3%) received second-line systemic treatment (Table 3).

Among the 210 patients with documented first-line treatment, 173 were evaluable for response, 12 were explicitly recorded as not evaluated, and response information was missing for 25. Among the 173 response-evaluable patients, complete response was observed in 9 patients, partial response in 72, stable disease in 56, and progressive disease in 36. The objective response rate was 81/173 (46.8%), and the disease control rate was 137/173 (79.2%) (Table 3).

### 3.5. Survival Outcomes in the Right-Sided Cohort

**Figure 2:**
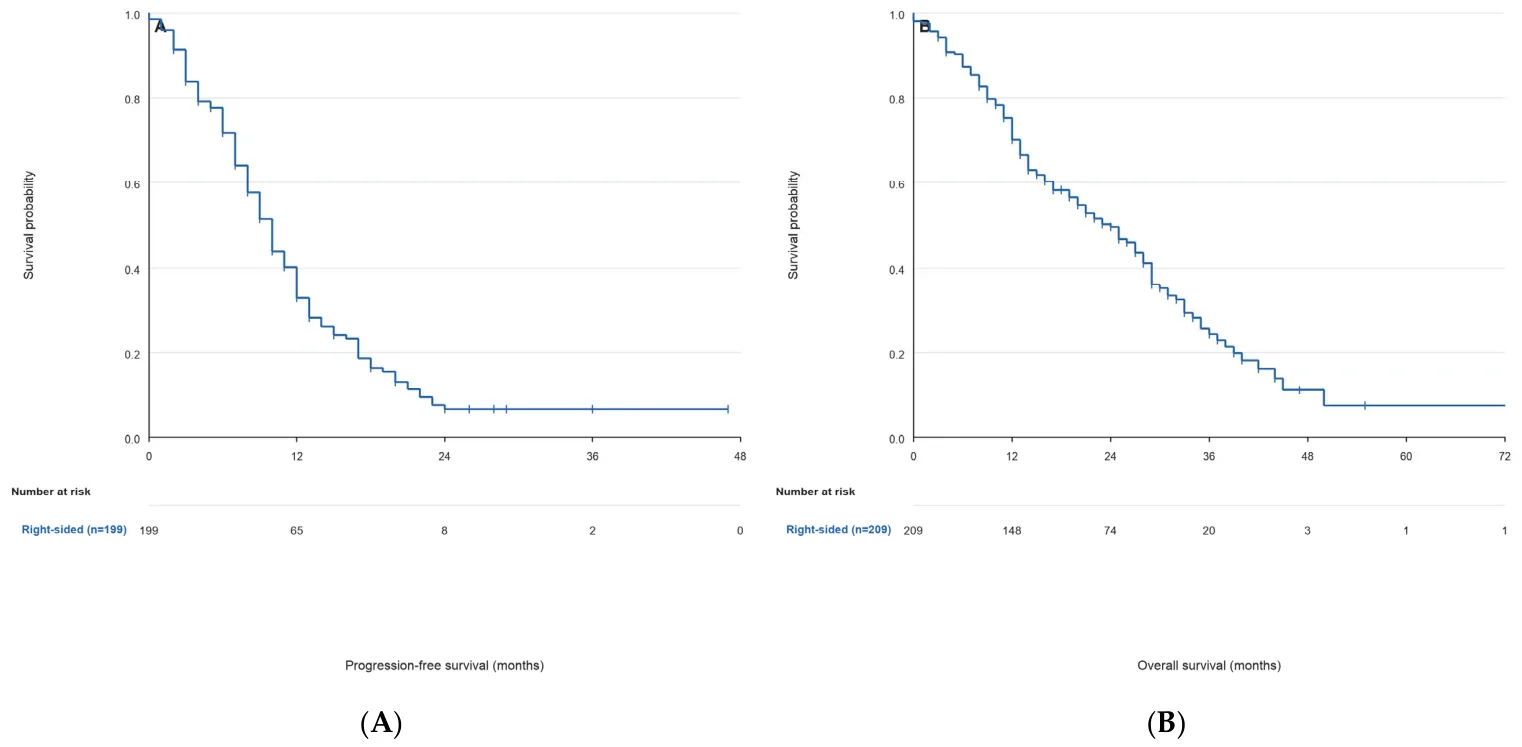
Kaplan–Meier survival curves in the right-sided cohort. (**A**) Progression-free survival among 199 evaluable patients, including 157 progression or death events. (**B**) Overall survival among 209 evaluable patients, including 135 deaths. Vertical tick marks indicate censored observations, and numbers at risk are presented at 12-month intervals. Estimates at later time points should be interpreted cautiously because few patients remained under observation.

Progression-free survival was evaluable in 199 patients, among whom 157 progression or death events were observed. The median PFS was 10.0 months (95% CI, 9.0–11.0). The estimated 12-month and 24-month PFS rates were 32.8% and 6.5%, respectively (Figure 2A).

Overall survival was evaluable in 209 patients, among whom 135 deaths were observed. The median OS was 24.0 months (95% CI, 19.0–28.0). The estimated 12-, 24-, and 36-month OS rates were 70.2%, 49.5%, and 24.3%, respectively (Figure 2B).

Using the reverse Kaplan–Meier method, median follow-up was 32.0 months (95% CI, 29.0–33.0) among the 663 patients with evaluable OS in the combined right- and left-sided colon cohort. Median follow-up was 32.0 months (95% CI, 27.0–34.0) among 209 patients with evaluable OS in the right-sided cohort and 32.0 months (95% CI, 29.0–34.0) among 454 patients with evaluable OS in the left-sided cohort.

### 3.6. Prognostic Factors for PFS and OS in the Right-Sided Cohort

Univariable Cox regression analyses are shown in Table 4. ECOG PS 2–4 was associated with inferior PFS (HR 2.07, 95% CI 1.16–3.69; *p* = 0.014) and inferior OS (HR 2.31, 95% CI 1.31–4.10; *p* = 0.004). Primary tumor resection was associated with longer OS (HR 0.50, 95% CI 0.32–0.81; *p* = 0.004), while metastasectomy was also associated with longer OS (HR 0.40, 95% CI 0.22–0.74; *p* = 0.004).

First-line anti-EGFR-based treatment was associated with longer OS compared with anti-VEGF-based treatment in univariable analysis (HR 0.64, 95% CI 0.44–0.93; *p* = 0.019), whereas the association with PFS was not statistically significant. Irinotecan-based chemotherapy was associated with inferior OS compared with oxaliplatin-based chemotherapy (HR 1.54, 95% CI 1.06–2.22; *p* = 0.023). Objective response to first-line therapy was strongly associated with longer PFS and OS; however, this was interpreted as an on-treatment outcome rather than a baseline prognostic factor.

Surgery timing relative to first-line treatment initiation was available for 149 patients who underwent primary tumor resection and 28 patients who underwent metastasectomy. Among these, 133 primary tumor resections and 10 metastasectomies occurred before first-line treatment, while 16 primary tumor resections and 18 metastasectomies occurred afterward. In the time-dependent sensitivity analysis, the association between primary tumor resection and OS was attenuated and was no longer statistically significant (HR 0.65, 95% CI 0.40–1.06; *p* = 0.087; 170 patients and 105 deaths). Metastasectomy remained associated with longer OS (HR 0.46, 95% CI 0.23–0.92; *p* = 0.028; 203 patients and 130 deaths). These results are presented in Supplementary Table S8.

### 3.7. Multivariable Analyses for Progression-Free Survival and Overall Survival

The exploratory complete-case multivariable PFS model included 80 patients and 62 progression or death events, corresponding to 12.4 events per included variable. None of the five covariates reached statistical significance. ECOG performance status 2–4 showed a nonsignificant association with poorer PFS (adjusted HR 2.12, 95% CI 0.94–4.79; *p* = 0.070), while the anti-EGFR versus anti-VEGF estimate was also nonsignificant (adjusted HR 0.76, 95% CI 0.42–1.37; *p* = 0.364) (Table 5). The primary multivariable Cox model included 88 patients and 54 deaths, corresponding to 10.8 events per included variable. Of the 213 patients in the right-sided cohort, 125 were not included in the complete-case model. The non-mutually exclusive factors contributing to exclusion were missing ECOG performance status in 85 patients, missing primary tumor resection status in 27, treatment outside the modeled anti-EGFR/anti-VEGF comparison or missing treatment information in 28, an unclassifiable or other chemotherapy backbone in 20, and incomplete OS time or vital-status information in four. Variable-level missingness is reported in Supplementary Table S1.

Compared with the 125 patients excluded from the complete-case model, the 88 included patients had a lower frequency of synchronous/de novo metastatic disease (53.4% vs. 76.0%; *p* < 0.001). Age, sex, metastatic burden, and the frequencies of liver, lung, lymph-node, and peritoneal metastases did not differ significantly. Median OS was 24.0 months in complete cases and 23.0 months in excluded patients (log-rank *p* = 0.606). The complete comparison is presented in Supplementary Table S2.

The multiple-imputation sensitivity analysis included 179 patients and 116 deaths. The pooled adjusted estimates were as follows: ECOG 2–4 versus 0–1, HR 1.77 (95% CI 0.92–3.41; *p* = 0.089); primary tumor resection, HR 0.50 (95% CI 0.29–0.87; *p* = 0.015); metastasectomy, HR 0.51 (95% CI 0.26–1.01; *p* = 0.053); anti-EGFR versus anti-VEGF treatment, HR 0.68 (95% CI 0.45–1.03; *p* = 0.066); and irinotecan- versus oxaliplatin-based chemotherapy, HR 1.39 (95% CI 0.94–2.07; *p* = 0.101). Thus, the anti-EGFR estimate remained statistically nonsignificant, while the chemotherapy-backbone association observed in the complete-case model was attenuated and no longer statistically significant. Complete-case and multiple-imputation estimates are compared in Supplementary Table S3.

### 3.8. Exploratory Treatment Comparison: Anti-EGFR Versus Anti-VEGF

In an exploratory analysis restricted to patients treated with first-line anti-EGFR- or anti-VEGF-based regimens, 87 patients received anti-EGFR-based treatment and 98 patients received anti-VEGF-based treatment.

Baseline characteristics according to first-line biological treatment are presented in Supplementary Table S4. Compared with the anti-VEGF group, patients in the anti-EGFR group were younger (median age, 59.5 versus 62.5 years; absolute standardized mean difference [SMD], 0.28), had a lower frequency of peritoneal metastasis (10.3% versus 19.4%; SMD, 0.26), and more frequently underwent metastasectomy (18.4% versus 10.2%; SMD, 0.24). Documented BRAF-mutant disease was also less frequent in the anti-EGFR group (2.3% versus 7.1%; SMD, 0.23), although molecular testing was incomplete. These differences indicate that biological treatment allocation was nonrandom and potentially affected by clinical selection.

Missingness according to first-line biological treatment is presented in Supplementary Table S7. None of the comparisons reached statistical significance; however, moderate standardized differences were observed for missing MSI status, histological grade, mucinous histology, baseline CEA, and baseline CA19-9. Given the limited treatment-group sample sizes, differential missingness cannot be excluded.

Among patients with documented maintenance-treatment status, maintenance therapy was administered to 23/85 patients (27.1%) in the first-line anti-EGFR group and 29/97 patients (29.9%) in the first-line anti-VEGF group. Among patients with documented second-line treatment status, second-line therapy was administered to 48/86 patients (55.8%) and 55/97 patients (56.7%), respectively.

OS and follow-up were evaluable in 86 of the 87 patients treated with anti-EGFR-based therapy and all 98 patients treated with anti-VEGF-based therapy. Median follow-up was 31.0 months (95% CI, 27.0–34.0) in the anti-EGFR group and 36.0 months (95% CI, 26.0 months–upper limit not estimable) in the anti-VEGF group.

**Figure 3:**
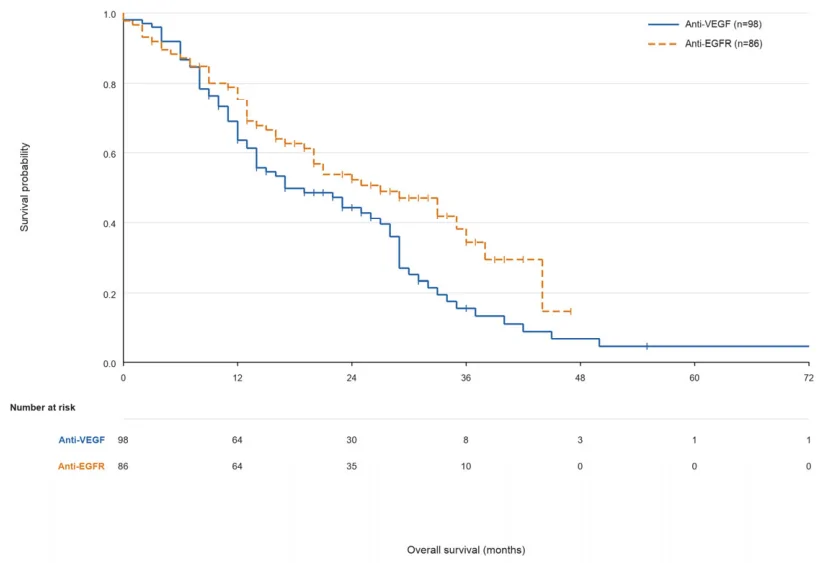
Kaplan–Meier overall survival according to first-line biological treatment. The analysis included 86 patients receiving anti-EGFR-based treatment, with 47 deaths, and 98 patients receiving anti-VEGF-based treatment, with 73 deaths (log-rank *p* = 0.022). Vertical tick marks indicate censored observations, and numbers at risk are presented at 12-month intervals. Late survival estimates should be interpreted cautiously because the risk sets became small after 36 months.

**Figure 4:**
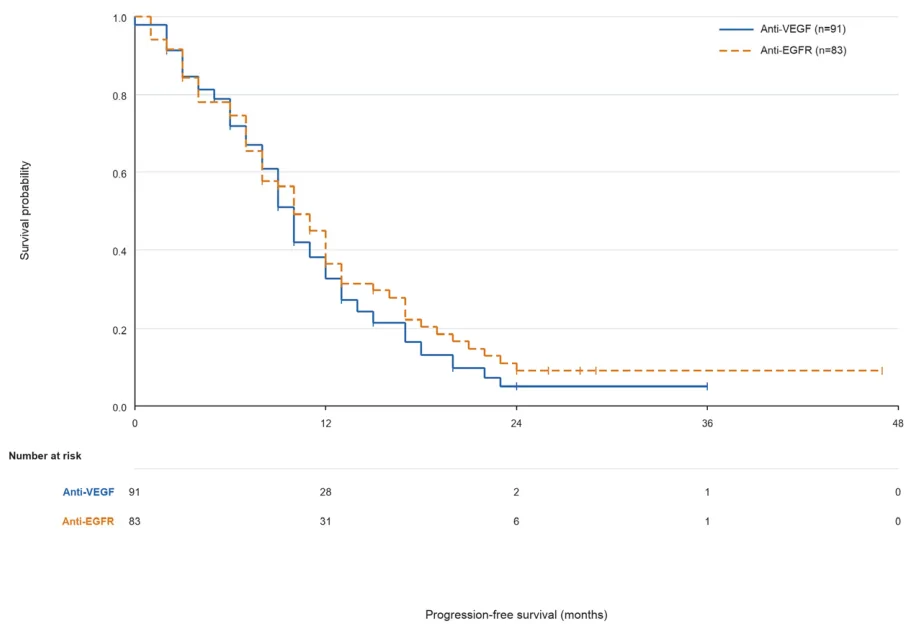
Kaplan–Meier progression-free survival according to first-line biological treatment. The analysis included 83 patients receiving anti-EGFR-based treatment, with 64 progression or death events, and 91 patients receiving anti-VEGF-based treatment, with 73 events (log-rank *p* = 0.370). Vertical tick marks indicate censored observations, and numbers at risk are presented at 12-month intervals. Estimates beyond 24 months should be interpreted cautiously because few patients remained at risk.

In unadjusted analysis, median OS was 27.0 months (95% CI, 19.0–36.0) in the anti-EGFR group and 17.0 months (95% CI, 14.0–27.0) in the anti-VEGF group (log-rank *p* = 0.022) (Figure 3). Median PFS was 10.0 months in both groups: 10.0 months (95% CI, 8.0–12.0) with anti-EGFR treatment and 10.0 months (95% CI, 8.0–11.0) with anti-VEGF treatment (log-rank *p* = 0.370) (Figure 4).

However, the anti-EGFR OS estimate was not statistically significant in the primary complete-case multivariable model (adjusted HR 0.61, 95% CI 0.32–1.15; *p* = 0.125) (Table 5), in the initial multiple-imputation sensitivity analysis (adjusted HR 0.68, 95% CI 0.45–1.03; *p* = 0.066) (Supplementary Table S3), or in the extended covariate-adjusted multiple-imputation model (adjusted HR 0.66, 95% CI 0.43–1.00; *p* = 0.051) (Supplementary Table S5). Accordingly, the Kaplan–Meier difference represents an unadjusted association between nonrandomized treatment groups and should not be interpreted as demonstrating a treatment effect.

The analysis-specific denominators differed because OS was evaluable in 86 anti-EGFR-treated and 98 anti-VEGF-treated patients, whereas PFS was evaluable in 83 and 91 patients, respectively. At 36 months, only 10 patients in the anti-EGFR group and 8 in the anti-VEGF group remained at risk for OS; by 48 months, no anti-EGFR-treated patients and only 3 anti-VEGF-treated patients remained at risk. Accordingly, separation of the curves at later time points should not be interpreted as reliable evidence of a treatment effect.

In a sensitivity analysis excluding the nine patients with documented BRAF-mutant disease, 204 patients remained in the right-sided cohort. Median OS remained 24.0 months (95% CI, 19.0–28.0), and median PFS remained 10.0 months (95% CI, 9.0–11.0). Among patients receiving anti-EGFR- or anti-VEGF-based treatment, median OS was 25.0 and 22.0 months, respectively (unadjusted HR 0.68, 95% CI 0.47–0.99; *p* = 0.043), while median PFS was 10.0 months in both groups (log-rank *p* = 0.439). In the repeated complete-case multivariable model, the anti-EGFR estimate remained statistically nonsignificant (adjusted HR 0.67, 95% CI 0.35–1.31; *p* = 0.242). These findings are presented in Supplementary Table S6.

### 3.9. Contextual Comparison with Left-Sided Colon Cancer

A contextual comparison between right-sided and left-sided colon cancer patients is shown in Table 6. Age and sex distribution were similar between groups. Synchronous/de novo metastatic disease was more common in left-sided colon cancer patients than in right-sided patients (78.6% vs. 66.7%; *p* = 0.001). BRAF mutation was more frequent in the right-sided cohort among tested patients (7.6% vs. 1.1%; *p* = 0.002). Liver metastasis was less frequent in right-sided tumors than in left-sided tumors (63.4% vs. 75.2%; *p* = 0.002), whereas lymph node metastasis was numerically more frequent in right-sided tumors (31.0% vs. 23.9%; *p* = 0.065).

First-line treatment selection differed substantially between groups. Anti-EGFR-based therapy was used less frequently in right-sided tumors than in left-sided tumors (40.8% vs. 66.2%), while anti-VEGF-based therapy was used more frequently in right-sided tumors (46.0% vs. 21.8%; *p* < 0.001).

**Figure 5:**
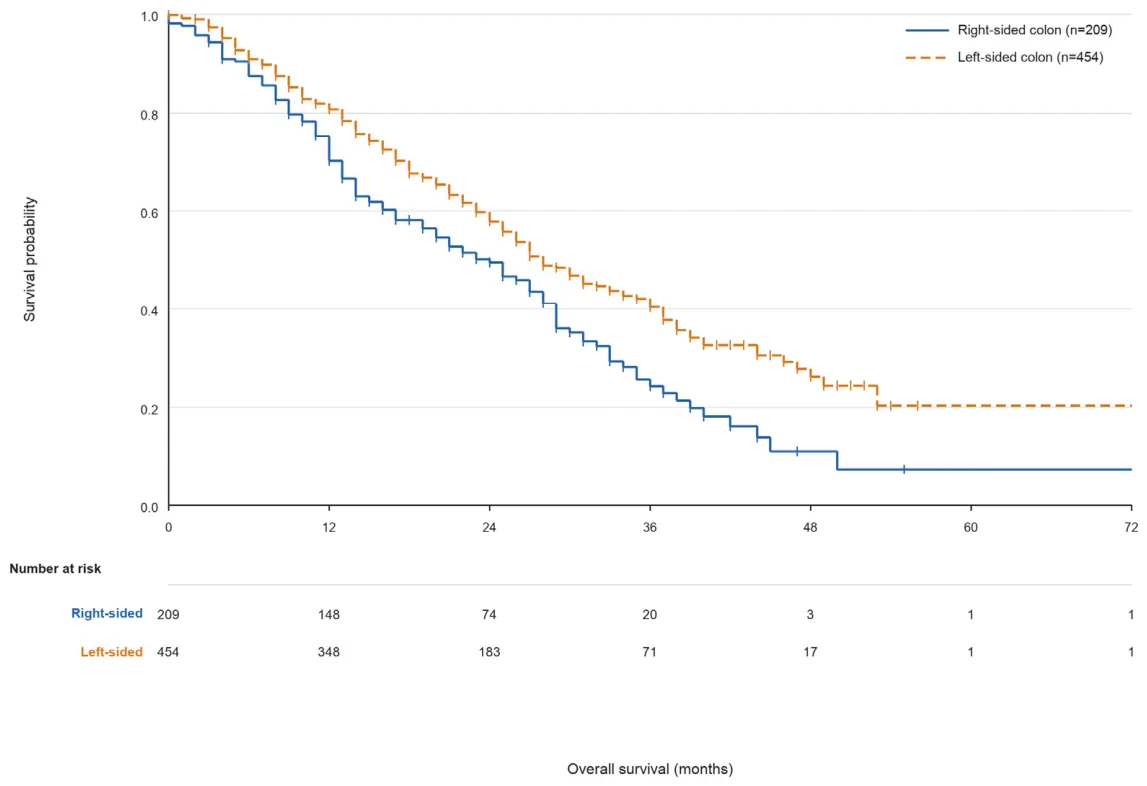
Kaplan–Meier overall survival comparison between right-sided and left-sided colon cancer. The analysis included 209 right-sided patients, with 135 deaths, and 454 left-sided patients, with 232 deaths (log-rank *p* < 0.001). Vertical tick marks indicate censored observations, and numbers at risk are presented at 12-month intervals. Estimates beyond 48 months should be interpreted cautiously because the risk sets were small.

Median PFS was numerically shorter in the right-sided group than in the left-sided group, but the difference was not statistically significant: 10.0 months versus 11.0 months (*p* = 0.244). Median OS was significantly shorter in right-sided colon cancer patients than in left-sided colon cancer patients: 24.0 months versus 28.0 months (HR 1.47, 95% CI 1.19–1.82; *p* < 0.001) (Figure 5).

## 4. Discussion

In this national 28-center registry analysis, we characterized the clinicopathologic presentation, metastatic distribution, treatment patterns, and outcomes of patients with right-sided RAS wild-type metastatic colon cancer treated in routine practice. The shorter OS observed relative to left-sided colon cancer was consistent with established sidedness literature and should be regarded as a confirmatory contextual finding rather than a novel demonstration of the prognostic importance of tumor location. Rectal primaries were excluded from this comparison. Although PFS did not differ significantly according to sidedness, OS was shorter in the right-sided cohort, possibly reflecting differences in disease biology, subsequent treatment opportunities, metastatic patterns, and access to local treatments over the complete disease course [9,10,11,15].

The unadjusted association between anti-EGFR treatment and longer OS contrasts with randomized-trial subgroup analyses and pooled evidence that generally support anti-VEGF-based strategies for right-sided RAS wild-type disease [9,11,15,16,17]. However, treatment allocation in this registry was not randomized. Anti-EGFR-treated patients were younger, had less frequent peritoneal involvement, and more frequently underwent metastasectomy, whereas documented BRAF-mutant disease was more frequent in the anti-VEGF group. Moreover, PFS was similar between the treatment groups, and the anti-EGFR estimate did not reach statistical significance in the complete-case, multiple-imputation, or extended covariate-adjusted OS models. The observed unadjusted OS difference is therefore more plausibly explained by clinical selection, adverse biological case mix, residual confounding, and post-baseline treatment differences than by comparative treatment effectiveness [20].

BRAF status was particularly relevant to this interpretation. After excluding the nine patients with documented BRAF-mutant disease, median OS increased from 17.0 to 22.0 months in the anti-VEGF group, while the repeated adjusted anti-EGFR estimate remained statistically nonsignificant. Nevertheless, BRAF testing was incomplete, preventing definitive assessment of its contribution. Contemporary first-line treatment of BRAF V600E-mutant metastatic colorectal cancer has also evolved following the BREAKWATER trial evaluating encorafenib, cetuximab, and mFOLFOX6 [21]. Because the present cohort was treated during 2016–2019, before this evidence and treatment strategy became available, the study cannot evaluate contemporary first-line BRAF-directed therapy.

Right-sided disease remains biologically heterogeneous, and the pooled evidence does not necessarily exclude a role for anti-EGFR therapy in every molecularly selected right-sided tumor. Anti-EGFR treatment may still be considered when rapid tumor shrinkage or conversion to resectability is an important objective, and ongoing molecular-hyperselection strategies may identify subgroups with greater potential benefit [12,13,14,18,22,23,24,25]. However, the present registry lacked sufficiently detailed molecular profiling, treatment intent information, tumor volume measurements, and subsequent-line regimen data to identify such a subgroup. Its findings should therefore be regarded as hypothesis-generating and should not alter treatment selection [25,26,27].

The metastatic and surgical findings also require cautious interpretation. Peritoneal and non-liver-dominant metastatic patterns may reduce opportunities for conversion therapy or metastasectomy [28,29,30,31,32]. In the time-dependent analysis, adjustment for surgical timing attenuated the primary-tumor-resection association, which was no longer statistically significant, whereas metastasectomy remained associated with longer OS. However, time-dependent modeling cannot eliminate selection according to patient fitness, treatment response, metastatic anatomy, tumor burden, or technical resectability. These surgical associations therefore remain noncausal [20].

The adjusted analyses were limited by missing data. The exploratory PFS and primary OS complete-case models included only 37.6% and 41.3% of the right-sided cohort, respectively, and the modeled and excluded populations differed in synchronous metastatic presentation. None of the covariates reached statistical significance in the exploratory PFS model. In the multiple-imputation OS analysis, the anti-EGFR estimate remained nonsignificant, while the chemotherapy-backbone association observed in the complete-case model was attenuated. Treatment response was strongly associated with survival, but it represents an on-treatment outcome rather than a baseline prognostic factor. These findings reinforce that the adjusted estimates are exploratory and cannot establish causality.

The study’s strengths include its national multicenter design, its focus on a dedicated right-sided RAS wild-type colon cancer cohort, and its description of treatment patterns extending beyond first-line selection to maintenance treatment, second-line treatment, and metastatic management. Excluding rectal primaries also improved the anatomical consistency of the contextual right-versus-left colon comparison.

Several limitations remain. The retrospective design introduced selection bias, missing data, residual confounding, and nonrandom treatment allocation. The de-identified analytic dataset did not retain patient-level identification of transverse colon tumors, preventing a sensitivity analysis based on an alternative anatomical classification. Detailed treatment exposure, dose intensity, treatment duration, discontinuation reasons, and subsequent-line regimens were unavailable. Response assessment was not centrally reviewed, and the registry did not document uniform application of RECIST criteria across participating centers; consequently, the reported response estimates may be affected by interinstitutional assessment variability. Reasons for individual missing values were not recorded, and incomplete ECOG, molecular, histopathologic, and tumor-marker data may have introduced selection bias and restricted biomarker-based interpretation. Microsatellite instability status was available in only 71 patients, of whom 15 were MSI-H. Because the cohort was treated between 2016 and 2019, the treatment era predates the adoption of first-line immune checkpoint inhibition for MSI-H metastatic colorectal cancer following KEYNOTE-177 [33]; these patients therefore received chemotherapy plus a biological agent accordingly, and the generalizability of the present findings to the current MSI-H standard of care is limited.

In conclusion, this study provides national multicenter real-world information on the presentation, management, and outcomes of right-sided RAS wild-type metastatic colon cancer. The unadjusted OS difference between anti-EGFR- and anti-VEGF-treated patients was not confirmed after measured covariate adjustment and cannot support comparative-effectiveness or causal inference. Prospective studies incorporating comprehensive molecular selection and contemporary treatment strategies are required to refine treatment decisions within this heterogeneous population.

## Figures and Tables

**Table 1:** Baseline clinicopathologic characteristics of patients with right-sided RAS wild-type metastatic colon cancer.

Variable	Right-Sided Cohort, *n* = 213
Age, median (range), years	61.5 (26–84)
Age data missing	1 (0.5%)
Sex	
Female	76 (35.7%)
Male	137 (64.3%)
Metastatic presentation	
Synchronous/de novo metastatic disease	142 (66.7%)
Metachronous metastatic disease	71 (33.3%)
ECOG performance status	
ECOG 0–1	110 (51.6%)
ECOG 2–4	18 (8.5%)
Missing	85 (39.9%)
BMI, median (IQR), kg/m^2^	26.1 (22.5–29.4)
BMI missing	53 (24.9%)
Smoking status	
Never smoker	59 (27.7%)
Current smoker	39 (18.3%)
Former smoker	20 (9.4%)
Missing	95 (44.6%)
BRAF mutation status	
Wild type	110 (51.6%)
Mutant	9 (4.2%)
Missing/not tested	94 (44.1%)
MSI status	
MSS	56 (26.3%)
MSI-H	15 (7.0%)
Missing/not tested	142 (66.7%)
Mucinous histology	
Non-mucinous	73 (34.3%)
Mucinous	21 (9.9%)
Missing	119 (55.9%)
Histological grade	
Grade 1–2	80 (37.6%)
Grade 3	28 (13.1%)
Missing	105 (49.3%)
Baseline CEA category	
Below threshold	11 (5.2%)
Above threshold	109 (51.2%)
Missing	93 (43.7%)
Baseline CA19-9 category	
Below threshold	50 (23.5%)
Above threshold	54 (25.4%)
Missing	109 (51.2%)
Primary tumor resection	
Yes	161 (75.6%)
No	25 (11.7%)
Missing	27 (12.7%)
Metastasectomy	
Yes	30 (14.1%)
No	183 (85.9%)

**Table 2:** Metastatic patterns in right-sided metastatic colon cancer.

Variable	*n* (%)
Metastatic site	
Liver metastasis	135 (63.4%)
Lung metastasis	45 (21.1%)
Peritoneal metastasis	33 (15.5%)
Lymph node metastasis	66 (31.0%)
Bone metastasis	6 (2.8%)
Brain metastasis	1 (0.5%)
Adrenal metastasis	5 (2.3%)
Other metastatic sites	12 (5.6%)
Liver-only metastasis	83 (39.0%)
Number of metastatic organs	
1 organ	139 (65.3%)
2 organs	57 (26.8%)
3 organs	16 (7.5%)
4 organs	1 (0.5%)

**Table 3:** First-line treatment patterns, maintenance and second-line treatment, and response outcomes.

Variable	*n* (%)
First-line treatment category	
Doublet + anti-VEGF	98 (46.0%)
Doublet + anti-EGFR	87 (40.8%)
Chemotherapy doublet without biologic	21 (9.9%)
Triplet-based treatment	4 (1.9%)
Missing/unclear	3 (1.4%)
Biological agent	
Bevacizumab-containing	102 (47.9%)
Cetuximab-containing	51 (23.9%)
Panitumumab-containing	36 (16.9%)
No biological agent/chemotherapy alone	21 (9.9%)
Missing/unclear	3 (1.4%)
Chemotherapy backbone	
Oxaliplatin-based	129 (60.6%)
Irinotecan-based	67 (31.5%)
Fluoropyrimidine-only/other	10 (4.7%)
Triplet	4 (1.9%)
Missing/unclear	3 (1.4%)
Maintenance treatment	
Yes	54 (25.4%)
No	153 (71.8%)
Missing	6 (2.8%)
Maintenance regimen among recipients (*n* = 54)	
Capecitabine + bevacizumab	16 (29.6%)
Capecitabine alone	10 (18.5%)
5-FU/leucovorin + bevacizumab	8 (14.8%)
5-FU/leucovorin + panitumumab	8 (14.8%)
5-FU/leucovorin + cetuximab	8 (14.8%)
Cetuximab alone	3 (5.6%)
Capecitabine + cetuximab	1 (1.9%)
Second-line treatment	
Yes	117 (54.9%)
No	91 (42.7%)
Missing	5 (2.3%)
Best response to first-line therapy	
Complete response	9 (4.2%)
Partial response	72 (33.8%)
Stable disease	56 (26.3%)
Progressive disease	36 (16.9%)
Not evaluated	12 (5.6%)
Missing	28 (13.1%)
Objective response rate, among evaluable patients	81/173 (46.8%)
Disease control rate, among evaluable patients	137/173 (79.2%)

Footnote: Unless otherwise specified, percentages were calculated using the complete right-sided cohort (*n* = 213). First-line treatment data were available for 210 patients; treatment information was missing or unclear for 3 patients. The 102 bevacizumab-containing regimens comprised 98 doublet-plus-anti-VEGF regimens and 4 triplet-plus-bevacizumab regimens. Maintenance-treatment status was available for 207 patients, and maintenance-regimen percentages were calculated among the 54 patients who received maintenance therapy. Second-line treatment status was available for 208 patients. Response was evaluable in 173 of the 210 treated patients; among the remaining 37 patients, 12 were documented as not evaluated and 25 had missing response data. The 28 missing responses shown using the complete-cohort denominator comprise these 25 patients plus the 3 patients with missing first-line treatment information. Maintenance-treatment and second-line-treatment percentages in this table use the full right-sided cohort as the denominator (*n* = 213), whereas the corresponding percentages in the text use the available-data denominators (54/207, 26.1% for maintenance treatment and 117/208, 56.3% for second-line treatment).

**Table 4:** Univariable Cox regression analysis for PFS and OS in the right-sided cohort.

Variable	PFS HR (95% CI)	*p* Value	OS HR (95% CI)	*p* Value
Age > 60 vs. ≤ 60 years	0.92 (0.67–1.26)	0.585	1.12 (0.79–1.58)	0.529
Male vs. female	1.20 (0.86–1.67)	0.274	1.07 (0.74–1.53)	0.730
Metachronous vs. synchronous disease	1.02 (0.73–1.43)	0.905	1.07 (0.74–1.54)	0.731
ECOG 2–4 vs. 0–1	2.07 (1.16–3.69)	0.014	2.31 (1.31–4.10)	0.004
BRAF mutant vs. wild-type	1.88 (0.90–3.94)	0.095	1.51 (0.61–3.79)	0.375
MSI-H vs. MSS	1.19 (0.60–2.34)	0.616	0.99 (0.41–2.42)	0.991
Mucinous vs. non-mucinous	0.82 (0.46–1.46)	0.502	0.86 (0.42–1.78)	0.687
Liver metastasis, yes vs. no	1.15 (0.83–1.61)	0.397	1.20 (0.84–1.72)	0.319
Peritoneal metastasis, yes vs. no	1.11 (0.74–1.69)	0.609	0.89 (0.56–1.43)	0.638
Lymph node metastasis, yes vs. no	0.92 (0.65–1.29)	0.619	0.97 (0.68–1.40)	0.888
≥2 metastatic organs vs. 1 organ	1.27 (0.92–1.76)	0.143	1.39 (0.99–1.96)	0.060
Liver-only metastasis, yes vs. no	0.89 (0.64–1.23)	0.468	0.89 (0.63–1.27)	0.532
Primary tumor resection, yes vs. no	0.63 (0.40–1.00)	0.050	0.50 (0.32–0.81)	0.004
Metastasectomy, yes vs. no	0.63 (0.39–1.04)	0.071	0.40 (0.22–0.74)	0.004
Anti-EGFR vs. anti-VEGF	0.86 (0.61–1.20)	0.367	0.64 (0.44–0.93)	0.019
Irinotecan vs. oxaliplatin backbone	1.37 (0.97–1.94)	0.075	1.54 (1.06–2.22)	0.023
Objective response vs. SD/PD	0.41 (0.29–0.59)	<0.001	0.52 (0.35–0.77)	0.001

**Table 5:** Complete-case multivariable Cox regression analyses for progression-free and overall survival.

Variable	PFS Adjusted HR (95% CI)	*p* Value	OS Adjusted HR (95% CI)	*p* Value
ECOG 2–4 vs. 0–1	2.12 (0.94–4.79)	0.070	1.81 (0.78–4.16)	0.165
Primary tumor resection, yes vs. no	0.62 (0.31–1.24)	0.174	0.39 (0.19–0.79)	0.009
Metastasectomy, yes vs. no	1.20 (0.58–2.51)	0.623	0.74 (0.30–1.83)	0.515
Anti-EGFR vs. anti-VEGF	0.76 (0.42–1.37)	0.364	0.61 (0.32–1.15)	0.125
Irinotecan- vs. oxaliplatin-based chemotherapy	1.24 (0.71–2.16)	0.455	2.00 (1.13–3.53)	0.018

Footnote: The PFS model included 80 patients and 62 progression or death events. The OS model included 88 patients and 54 deaths. PFS, progression-free survival; OS, overall survival; HR, hazard ratio; CI, confidence interval.

**Table 6:** Contextual comparison of right-sided and left-sided colon cancer patients.

Variable	Right-Sided Colon, *n* = 213	Left-Sided Colon, *n* = 464	*p* Value
Age, median (range), years	61.5 (26–84)	61.0 (23–88)	0.651
Male sex	137 (64.3%)	298 (64.2%)	1.000
Synchronous/de novo metastatic disease	142 (66.7%)	364/463 (78.6%)	0.001
ECOG 2–4 among known ECOG	18/128 (14.1%)	23/261 (8.8%)	0.159
BRAF mutant among tested patients	9/119 (7.6%)	3/278 (1.1%)	0.002
MSI-H among tested patients	15/71 (21.1%)	28/158 (17.7%)	0.669
Grade 3 among known grade	28/108 (25.9%)	34/225 (15.1%)	0.026
Liver metastasis	135 (63.4%)	349 (75.2%)	0.002
Lung metastasis	45 (21.1%)	103 (22.2%)	0.831
Peritoneal metastasis	33 (15.5%)	67 (14.4%)	0.809
Lymph node metastasis	66 (31.0%)	111 (23.9%)	0.065
First-line anti-EGFR treatment	87 (40.8%)	307 (66.2%)	<0.001
First-line anti-VEGF treatment	98 (46.0%)	101 (21.8%)	<0.001
Median PFS, months	10.0	11.0	0.244
Median OS, months	24.0	28.0	<0.001
Median follow-up, months (95% CI)	32.0 (27.0–34.0)	32.0 (29.0–34.0)	—

## Data Availability

The data that support the findings of this study are derived from the ONKO-KOLON Türkiye Registry. The datasets are not publicly available because they contain potentially identifiable patient information and are subject to ethical and institutional restrictions. De-identified data may be made available from the corresponding author upon reasonable request and with permission from the ONKO-KOLON Turkey Registry Steering Committee and the relevant Ethics Committee, where applicable.

## Supplementary Materials

The following supporting information can be downloaded at: https://www.mdpi.com/article/10.3390/clinpract16090158/s1, Figure S1. Patient selection and analysis-specific populations. PFS, progression-free survival; OS, overall survival; MSI, microsatellite instability; Figure S2. Kaplan–Meier survival curves in the right-sided cohort. (**A**) Progression-free survival among 199 evaluable patients, including 157 progression or death events. (**B**) Overall survival among 209 evaluable patients, including 135 deaths. Vertical tick marks indicate censored observations, and numbers at risk are presented at 12-month intervals. Estimates at later time points should be interpreted cautiously because few patients remained under observation; Figure S3. Kaplan–Meier overall survival according to first-line biological treatment. The analysis included 86 patients receiving anti-EGFR-based treatment, with 47 deaths, and 98 patients receiving anti-VEGF-based treatment, with 73 deaths (log-rank *p* = 0.022). Vertical tick marks indicate censored observations, and numbers at risk are presented at 12-month intervals. Late survival estimates should be interpreted cautiously because the risk sets became small after 36 months; Figure S4. Kaplan–Meier progression-free survival according to first-line biological treatment. The analysis included 83 patients receiving anti-EGFR-based treatment, with 64 progression or death events, and 91 patients receiving anti-VEGF-based treatment, with 73 events (log-rank *p* = 0.370). Vertical tick marks indicate censored observations, and numbers at risk are presented at 12-month intervals. Estimates beyond 24 months should be interpreted cautiously because few patients remained at risk; Figure S5. Kaplan–Meier overall survival comparison between right-sided and left-sided colon cancer. The analysis included 209 right-sided patients, with 135 deaths, and 454 left-sided patients, with 232 deaths (log-rank *p* < 0.001). Vertical tick marks indicate censored observations, and numbers at risk are presented at 12-month intervals. Estimates beyond 48 months should be interpreted cautiously because the risk sets were small; Table S1. Availability and missingness of variables in the right-sided RAS wild-type metastatic colon cancer cohort; Table S2. Comparison of patients included in and excluded from the primary complete-case multivariable overall-survival model; Table S3. Primary complete-case and multiple-imputation sensitivity models for overall survival; Table S4. Baseline characteristics according to first-line biological treatment group; Table S5. Extended covariate-adjusted multiple-imputation model for overall survival; Table S6. Sensitivity analyses excluding patients with documented BRAF-mutant disease; Table S7. Missingness of key variables according to first-line biological treatment group; Table S8. Conventional and time-dependent sensitivity analyses of surgical exposures and overall survival.
