# Supplementary material for: Clinical Characteristics, Treatment Patterns, and Survival Outcomes of Right-Sided RAS/RAF Wild-Type Metastatic Colorectal Cancer: A Real-World Multicenter Cohort Study
Source: Clin Pract. 2026 Aug 24;16(9):158. doi: 10.3390/clinpract16090158 (PMC13605574; doi:10.3390/clinpract16090158)
Supplement: Supplementary file 1 [file clinpract-16-00158-s001.zip › clinpract-4336835-Supplementary Tables.pdf]

SUPPLEMENTARY MATERIALS

Clinical Characteristics, Treatment Patterns, and Survival Outcomes of Right-Sided RAS Wild-Type Metastatic Colorectal Cancer: A Real-World Multicenter Cohort Study

Nur Deniz Yildiz, Çağatay Arslan, İlker Nihat Okten, Umut Kefeli, Mahmut Emre Yildirim, Nuri Karadurmus, Tuba Baydas, Bülent Karabulut, Irfan Cicin, Cemil Bilir, Melike Ozcelik, Timucin Cil, Sinemis Celik, Oktay Bozkurt, Hakan Harputluoglu, Bala Başak Oven, Mehmet Artaç, Hacı Mehmet Turk, Ahmet Alacacıoğlu, Mahmut Gumus, and Suayib Yalcin

Clinics and Practice | Manuscript ID: clinpract-4336835

Contents

Item	Title
Table S1	Availability and missingness of variables in the right-sided RAS wild-type metastatic colon cancer cohort
Table S2	Comparison of patients included in and excluded from the primary complete-case multivariable overall-survival model
Table S3	Primary complete-case and multiple-imputation sensitivity models for overall survival
Table S4	Baseline characteristics according to first-line biological treatment group
Table S5	Extended covariate-adjusted multiple-imputation model for overall survival
Table S6	Sensitivity analyses excluding patients with documented BRAF-mutant disease
Table S7	Missingness of key variables according to first-line biological treatment group
Table S8	Conventional and time-dependent sensitivity analyses of surgical exposures and overall survival

Table S1. Availability and missingness of variables in the right-sided RAS wild-type metastatic colon cancer cohort

Variable	Denominator	Available/evaluable, n (%)	Missing/not documented, n (%)
Age	213	212 (99.5%)	1 (0.5%)
Sex	213	213 (100.0%)	0 (0.0%)
Synchronous/metachronous presentation	213	213 (100.0%)	0 (0.0%)
Number of metastatic organs	213	213 (100.0%)	0 (0.0%)
ECOG performance status	213	128 (60.1%)	85 (39.9%)
Body mass index	213	160 (75.1%)	53 (24.9%)
Smoking status	213	118 (55.4%)	95 (44.6%)
BRAF mutation status	213	119 (55.9%)	94 (44.1%)
MSI status	213	71 (33.3%)	142 (66.7%)
Mucinous histology	213	94 (44.1%)	119 (55.9%)
Histological grade	213	108 (50.7%)	105 (49.3%)
Baseline CEA category	213	120 (56.3%)	93 (43.7%)
Baseline CA19-9 category	213	104 (48.8%)	109 (51.2%)
Primary tumor resection	213	186 (87.3%)	27 (12.7%)
Metastasectomy	213	213 (100.0%)	0 (0.0%)
First-line treatment	213	210 (98.6%)	3 (1.4%)
Treatment response (among 210 with treatment data)	210	173 (82.4%)	Not evaluated: 12 (5.7%); missing: 25 (11.9%)
Progression-free survival	213	199 (93.4%)	14 (6.6%)
Overall survival	213	209 (98.1%)	4 (1.9%)
Multivariable OS complete-case population	213	88 (41.3%)	125 (58.7%)

Note. Percentages use the denominator shown. Unless otherwise specified, the denominator is the complete right-sided cohort (N = 213). Treatment-response availability is summarized among the 210 patients with documented first-line treatment.

ECOG, Eastern Cooperative Oncology Group; BRAF, B-Raf proto-oncogene; MSI, microsatellite instability; CEA, carcinoembryonic antigen; CA19-9, carbohydrate antigen 19-9; PFS, progression-free survival; OS, overall survival.

Table S2. Comparison of patients included in and excluded from the primary complete-case multivariable overall-survival model

Characteristic	Category	Complete-case model (n = 88)	Excluded from model (n = 125)	p-value
**Age, median (IQR), years**		62.0 (54.0–69.0)	61.0 (53.0–67.0)	0.548
**Male sex**		51 (58.0%)	86 (68.8%)	0.112
**Synchronous/de novo metastatic disease**		47 (53.4%)	95 (76.0%)	<0.001
**Two or more metastatic organs**		27 (30.7%)	47 (37.6%)	0.310
**Liver metastasis**		51 (58.0%)	84 (67.2%)	0.194
**Lung metastasis**		19 (21.6%)	26 (20.8%)	1.000
**Lymph-node metastasis**		25 (28.4%)	41 (32.8%)	0.549
**Peritoneal metastasis**		16 (18.2%)	17 (13.6%)	0.442
**Metastasectomy**		15 (17.0%)	15 (12.0%)	0.322
**ECOG performance status**	ECOG 0–1	78 (88.6%)	32 (25.6%)	<0.001
	ECOG 2–4	10 (11.4%)	8 (6.4%)	
	Missing	0 (0.0%)	85 (68.0%)	
**BRAF mutation status**	Wild type	44 (50.0%)	66 (52.8%)	0.660
	Mutant	5 (5.7%)	4 (3.2%)	
	Missing/not tested	39 (44.3%)	55 (44.0%)	
**MSI status**	MSS	31 (35.2%)	25 (20.0%)	0.043
	MSI-H	6 (6.8%)	9 (7.2%)	
	Missing/not tested	51 (58.0%)	91 (72.8%)	
**Primary tumor resection**	Yes	75 (85.2%)	86 (68.8%)	<0.001
	No	13 (14.8%)	12 (9.6%)	
	Missing	0 (0.0%)	27 (21.6%)	

Characteristic	Category	Complete-case model (n = 88)	Excluded from model (n = 125)	p-value
**First-line treatment category**	Anti-VEGF	45 (51.1%)	53 (42.4%)	<0.001
	Anti-EGFR	43 (48.9%)	44 (35.2%)	
	Triplet	0 (0.0%)	4 (3.2%)	
	Doublet without biologic	0 (0.0%)	21 (16.8%)	
	Missing	0 (0.0%)	3 (2.4%)	
**Chemotherapy backbone**	Oxaliplatin	49 (55.7%)	80 (64.0%)	<0.001
	Irinotecan	39 (44.3%)	25 (20.0%)	
	Other/unclassifiable	0 (0.0%)	20 (16.0%)	
**Median OS, months (Kaplan–Meier)**		24.0	23.0	0.606

Note. Values are n (%) unless otherwise specified. Percentages use the complete-case (n = 88) or excluded (n = 125) group as the denominator and therefore include missing categories where displayed.

Age was compared using the Mann-Whitney U test. Binary variables were compared using Fisher's exact test; multilevel categorical variables were compared using the chi-square test. OS distributions were compared using the log-rank test. The displayed p-value for a multilevel variable refers to the overall distribution.

IQR, interquartile range; ECOG, Eastern Cooperative Oncology Group; BRAF, B-Raf proto-oncogene; MSI-H, microsatellite instability-high; MSS, microsatellite stable; VEGF, vascular endothelial growth factor; EGFR, epidermal growth factor receptor; OS, overall survival.

Table S3. Primary complete-case and multiple-imputation sensitivity models for overall survival

Variable	Complete-case model, adjusted HR (95% CI)	Complete-case p-value	Multiple-imputation sensitivity, adjusted HR (95% CI)	Multiple-imputation p-value
ECOG 2–4 vs 0–1	1.81 (0.78–4.16)	0.165	1.77 (0.92–3.41)	0.089
Primary tumor resection, yes vs no	0.39 (0.19–0.79)	0.009	0.50 (0.29–0.87)	0.015
Metastasectomy, yes vs no	0.74 (0.30–1.83)	0.515	0.51 (0.26–1.01)	0.053
Anti-EGFR vs anti-VEGF	0.61 (0.32–1.15)	0.125	0.68 (0.45–1.03)	0.066
Irinotecan vs oxaliplatin backbone	2.00 (1.13–3.53)	0.018	1.39 (0.94–2.07)	0.101

Note. The complete-case model included 88 patients and 54 deaths. The multiple-imputation sensitivity population included 179 patients and 116 deaths. Fifty imputed datasets were generated by chained equations with predictive mean matching.

Within the multiple-imputation population, missing ECOG performance status (n = 68) and primary tumor resection status (n = 25) were imputed; age (missing in one patient) was included as an auxiliary predictor. Estimates were pooled using Rubin's rules.

HR, hazard ratio; CI, confidence interval; ECOG, Eastern Cooperative Oncology Group; EGFR, epidermal growth factor receptor; VEGF, vascular endothelial growth factor.

Table S4. Baseline characteristics according to first-line biological treatment group

Characteristic	Category	Anti-EGFR (n = 87)	Anti-VEGF (n = 98)	Absolute SMD
**Age, median (IQR), years**		59.5 (49.0–66.8)	62.5 (55.2–69.0)	0.28
**Male sex**		55 (63.2%)	64 (65.3%)	0.04
**Synchronous/de novo metastatic disease**		59 (67.8%)	61 (62.2%)	0.12
**Two or more metastatic organs**		27 (31.0%)	36 (36.7%)	0.12
**Liver metastasis**		57 (65.5%)	61 (62.2%)	0.07
**Liver-only metastatic disease**		37 (42.5%)	37 (37.8%)	0.10
**Lung metastasis**		17 (19.5%)	21 (21.4%)	0.05
**Lymph-node metastasis**		28 (32.2%)	30 (30.6%)	0.03
**Peritoneal metastasis**		9 (10.3%)	19 (19.4%)	0.26
**Metastasectomy**		16 (18.4%)	10 (10.2%)	0.24
**ECOG performance status**	ECOG 0–1	49 (56.3%)	50 (51.0%)	0.11
	ECOG 2–4	6 (6.9%)	11 (11.2%)	0.15
	Missing	32 (36.8%)	37 (37.8%)	0.02
**Primary tumor resection**	Yes	62 (71.3%)	75 (76.5%)	0.12
	No	12 (13.8%)	11 (11.2%)	0.08
	Missing	13 (14.9%)	12 (12.2%)	0.08
**BRAF mutation status**	Wild type	49 (56.3%)	47 (48.0%)	0.17
	Mutant	2 (2.3%)	7 (7.1%)	0.23
	Missing/not tested	36 (41.4%)	44 (44.9%)	0.07
**MSI status**	MSS	20 (23.0%)	31 (31.6%)	0.19
	MSI-H	4 (4.6%)	8 (8.2%)	0.15
	Missing/not tested	63 (72.4%)	59 (60.2%)	0.26

Characteristic	Category	Anti-EGFR (n = 87)	Anti-VEGF (n = 98)	Absolute SMD
**Chemotherapy backbone**	Oxaliplatin-based	54 (62.1%)	62 (63.3%)	0.02
	Irinotecan-based	32 (36.8%)	32 (32.7%)	0.09
	Other/unclassifiable	1 (1.1%)	4 (4.1%)	0.18

Note. Values are n (%) unless otherwise specified. Percentages use the full anti-EGFR (n = 87) or anti-VEGF (n = 98) treatment group as the denominator, including missing categories where displayed.

The absolute standardized mean difference (SMD) was used to describe covariate imbalance independently of sample size; values of 0.20 or greater were considered potentially meaningful.

IQR, interquartile range; SMD, standardized mean difference; ECOG, Eastern Cooperative Oncology Group; BRAF, B-Raf proto-oncogene; MSI-H, microsatellite instability-high; MSS, microsatellite stable; EGFR, epidermal growth factor receptor; VEGF, vascular endothelial growth factor.

Table S5. Extended covariate-adjusted multiple-imputation model for overall survival

Variable	Adjusted HR (95% CI)	p-value
Anti-EGFR vs anti-VEGF	0.66 (0.43–1.00)	0.051
Age, per 10-year increase	0.92 (0.77–1.09)	0.330
Male vs female	1.28 (0.83–1.97)	0.264
ECOG 2–4 vs 0–1	2.05 (1.05–4.00)	0.035
Synchronous vs metachronous presentation	0.64 (0.39–1.04)	0.073
Two or more vs one metastatic organ	1.36 (0.89–2.08)	0.154
Liver metastasis, yes vs no	1.16 (0.72–1.86)	0.538
Peritoneal metastasis, yes vs no	0.93 (0.50–1.74)	0.820
Primary tumor resection, yes vs no	0.44 (0.24–0.82)	0.010
Metastasectomy, yes vs no	0.51 (0.25–1.03)	0.059
Irinotecan vs oxaliplatin backbone	1.26 (0.82–1.94)	0.286

Note. The extended model included 179 patients and 116 deaths and contained 11 covariates (10.5 deaths per covariate). Fifty imputed datasets were generated, and estimates were pooled using Rubin's rules.

This model was an exploratory sensitivity analysis intended to examine the stability of the anti-EGFR estimate after broader measured-covariate adjustment. It does not eliminate residual confounding and should not be interpreted as evidence of comparative treatment effectiveness.

HR, hazard ratio; CI, confidence interval; ECOG, Eastern Cooperative Oncology Group; EGFR, epidermal growth factor receptor; VEGF, vascular endothelial growth factor.

Table S6. Sensitivity analyses excluding patients with documented BRAF-mutant disease

Analysis	Anti-EGFR / estimate	Anti-VEGF / comparator	Effect estimate	p-value
Right-sided cohort OS	n=200; deaths=130	—	Median OS, months (95% CI): 24.0 (19.0–28.0)	—
Right-sided cohort PFS	n=190; events=149	—	Median PFS, months (95% CI): 10.0 (9.0–11.0)	—
Unadjusted OS by first-line biological treatment	n=84; deaths=47; median 25.0 (19.0–36.0) months	n=91; deaths=68; median 22.0 (14.0–28.0) months	HR (95% CI): 0.68 (0.47–0.99)	0.043
Unadjusted PFS by first-line biological treatment	n=81; events=62; median 10.0 (8.0–12.0) months	n=84; events=67; median 10.0 (9.0–12.0) months	Log-rank comparison	0.439
Primary complete-case adjusted OS model	n=83; deaths=51	Reference	Adjusted HR (95% CI): 0.67 (0.35–1.31)	0.242

Note. Nine patients with documented BRAF-mutant disease were excluded, leaving 204 patients in the right-sided cohort. Patients with missing BRAF results were retained; therefore, this sensitivity population should not be interpreted as a confirmed BRAF wild-type cohort.

The treatment-specific OS hazard ratio compares anti-EGFR with anti-VEGF treatment. The adjusted model used the same five covariates as the primary complete-case OS model.

BRAF, B-Raf proto-oncogene; OS, overall survival; PFS, progression-free survival; HR, hazard ratio; CI, confidence interval; EGFR, epidermal growth factor receptor; VEGF, vascular endothelial growth factor.

Table S7. Missingness of key variables according to first-line biological treatment group

Variable	Missing in anti-EGFR (n = 87), n (%)	Missing in anti-VEGF (n = 98), n (%)	Absolute SMD	p-value
ECOG performance status	32 (36.8%)	37 (37.8%)	0.02	1.000
BRAF mutation status	36 (41.4%)	44 (44.9%)	0.07	0.658
MSI status	63 (72.4%)	59 (60.2%)	0.26	0.089
Histological grade	47 (54.0%)	40 (40.8%)	0.27	0.079
Mucinous histology	53 (60.9%)	48 (49.0%)	0.24	0.107
Baseline CEA category	33 (37.9%)	48 (49.0%)	0.22	0.140
Baseline CA19-9 category	39 (44.8%)	57 (58.2%)	0.27	0.078

Note. The analysis included patients receiving first-line anti-EGFR (n = 87) or anti-VEGF (n = 98) treatment. Missingness was defined as an absent or blank registry value.

P-values were calculated using Fisher's exact test. The absolute SMD was calculated for the binary missing-versus-available indicator; values of 0.20 or greater were considered potentially meaningful. None of the individual comparisons reached statistical significance.

SMD, standardized mean difference; ECOG, Eastern Cooperative Oncology Group; BRAF, B-Raf proto-oncogene; MSI, microsatellite instability; CEA, carcinoembryonic antigen; CA19-9, carbohydrate antigen 19-9; EGFR, epidermal growth factor receptor; VEGF, vascular endothelial growth factor.

Table S8. Conventional and time-dependent sensitivity analyses of surgical exposures and overall survival

Surgical exposure	Model	Patients	Deaths	HR (95% CI)	p-value
Primary tumor resection	Conventional baseline-classified univariable Cox model	183	115	0.50 (0.32–0.81)	0.004
Primary tumor resection	Time-dependent univariable Cox sensitivity model	170	105	0.65 (0.40–1.06)	0.087
Metastasectomy	Conventional baseline-classified univariable Cox model	209	135	0.40 (0.22–0.74)	0.004
Metastasectomy	Time-dependent univariable Cox sensitivity model	203	130	0.46 (0.23–0.92)	0.028

Note. Conventional rows reproduce the primary SPSS univariable estimates reported in Table 4 of the manuscript, with surgery classified as an ever/never baseline exposure. In time-dependent models, exposure changed from unexposed to exposed on the recorded surgery date, with first-line treatment initiation as time zero.

For primary tumor resection, the time-dependent model included 170 patients, 105 deaths, and 186 start-stop intervals: 131 exposures occurred before baseline and 16 after baseline; 10 exposed patients were excluded because timing was unavailable. For metastasectomy, the model included 203 patients, 130 deaths, and 221 intervals: 9 exposures occurred before baseline and 18 after baseline; 2 exposed patients were excluded because timing was unavailable. No exposure occurred after the end of observed follow-up in either model.

In the full descriptive timing data, 133 of 149 primary tumor resections and 10 of 28 metastasectomies occurred before first-line treatment; the remaining 16 resections and 18 metastasectomies occurred afterward. Model audit counts differ slightly because time-dependent analysis additionally required evaluable positive OS and complete timing information.

Models were univariable and exploratory. HR, hazard ratio; CI, confidence interval; OS, overall survival.
