# Supplementary material for: Clinical Characteristics, Treatment Patterns, and Survival Outcomes of Right-Sided RAS/RAF Wild-Type Metastatic Colorectal Cancer: A Real-World Multicenter Cohort Study
Source: Clin Pract. 2026 Aug 24;16(9):158. doi: 10.3390/clinpract16090158 (PMC13605574; doi:10.3390/clinpract16090158)

Figure 1. Patient selection flowchart

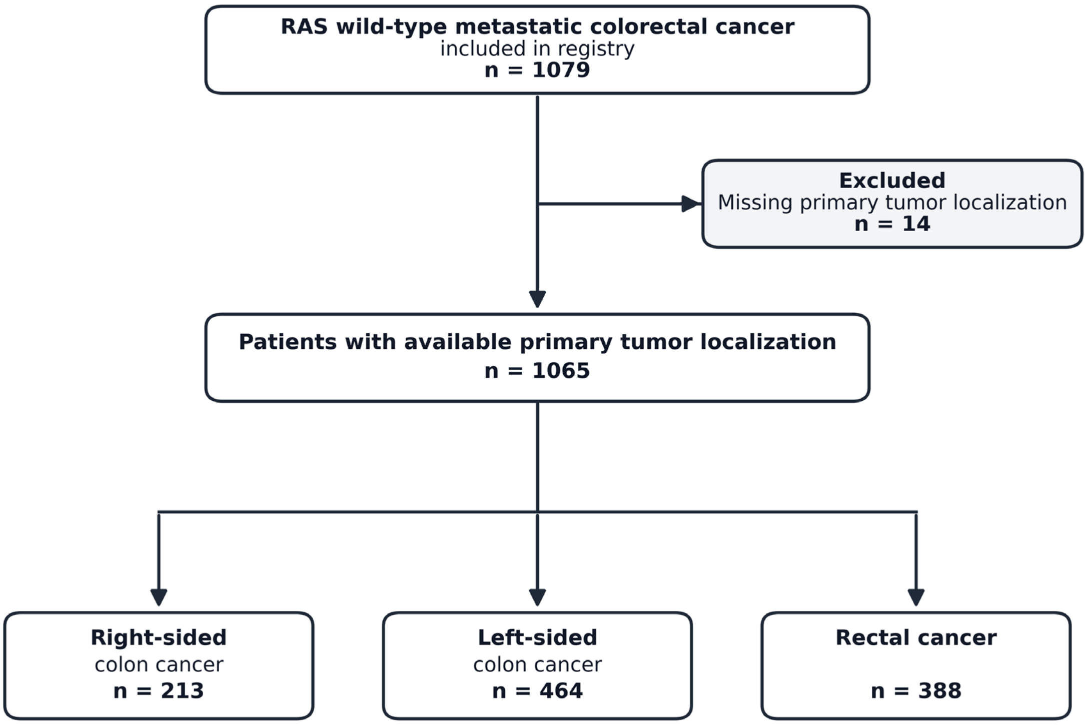

Supplement: Supplementary file 1 [file clinpract-16-00158-s001.zip › Figure_S1.pdf]
