# Supplementary material for: Clinical Characteristics, Treatment Patterns, and Survival Outcomes of Right-Sided RAS/RAF Wild-Type Metastatic Colorectal Cancer: A Real-World Multicenter Cohort Study
Source: Clin Pract. 2026 Aug 24;16(9):158. doi: 10.3390/clinpract16090158 (PMC13605574; doi:10.3390/clinpract16090158)

Figure 2. Kaplan–Meier survival curves in the right-sided cohort

Figure 2A. Progression-free survival in right-sided RAS wild-type metastatic colon cancer.

Figure 2B. Overall survival in right-sided RAS wild-type metastatic colon cancer.

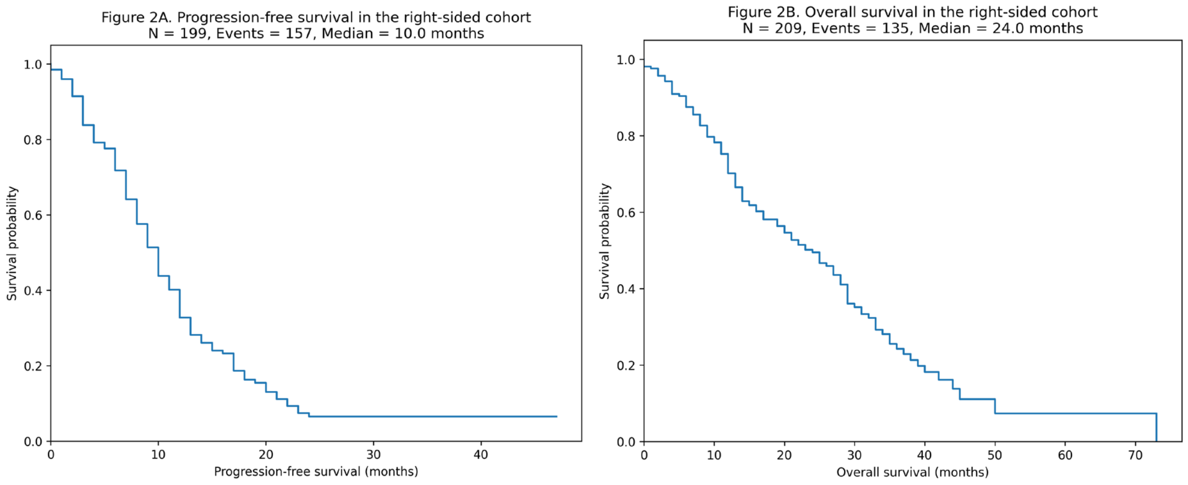

Supplement: Supplementary file 1 [file clinpract-16-00158-s001.zip › Figure_S2.pdf]
